# Draft Genome Sequences of Two Extensively Drug-Resistant Strains of *Mycobacterium tuberculosis* Belonging to the Euro-American S Lineage

**DOI:** 10.1128/genomeA.01771-15

**Published:** 2016-03-03

**Authors:** Lesibana A. Malinga, Thomas Abeel, Christopher A. Desjardins, Talent C. Dlamini, Gail Cassell, Sinéad B. Chapman, Bruce W. Birren, Ashlee M. Earl, Martie van der Walt

**Affiliations:** a South African Medical Research Council TB Platform, Pretoria, Gauteng, South Africa; b Department of Internal Medicine, University of Pretoria, Pretoria, Gauteng, South Africa; c Broad Institute, Cambridge, Massachusetts, USA; d Delft Bioinformatics Laboratory, Delft University of Technology, Delft, The Netherlands; e Medical Laboratory Sciences Department, Southern Africa Nazarene University, Manzini, Swaziland; f Harvard Medical School and Infectious Diseases Research Institute, Boston, Massachusetts, USA

## Abstract

We report the whole-genome sequencing of two extensively drug-resistant tuberculosis strains belonging to the Euro-American S lineage. The RSA 114 strain showed single-nucleotide polymorphisms predicted to have drug efflux activity.

## GENOME ANNOUNCEMENT

Drug-resistant tuberculosis (TB) caused by *Mycobacterium tuberculosis* is a global threat and a major public health problem in several countries (1). In South Africa, circulating *M. tuberculosis* strains are diverse (2), with three spoligotypes being most common: the Beijing spoligotype predominant in Western Cape, and the Euro-American LAM4 and S spoligotypes predominant in Gauteng and KwaZulu-Natal (KZN) provinces (3). The Euro-American S spoligotype (ST34) is prevalent in TB patients within KZN and Gauteng provinces (4).

We describe the draft genome sequences of two extensively drug-resistant (XDR) TB clinical strains of *M. tuberculosis* belonging to ST34. Permission to use these strains was granted by the University of Pretoria, Faculty of Health Sciences, Research Ethics Committee (206/2012). Both strains, RSA184 and RSA114, were isolated from patients from Swaziland. Spoligotyping and drug susceptibility testing (DST) were performed per standard protocols (5, 6).

DNA was extracted from heat-killed *M. tuberculosis* grown on slants, using a previously described chemical method (7). Illumina sequencing libraries were prepared as previously described (8) and sequenced using the Illumina HiSeq platform at the Broad Institute (Cambridge, MA, USA). Reads from RSA114 and RSA184 were assembled into draft genomes using ALLPATHS-LG with Pilon (9). The genome assemblies of 4,416,700 bp and 4,389,272 bp for RSA114 and RSA 184, respectively, were annotated by aligning each assembly to the H37Rv genome (CP003248.2) using Nucmer (10). For those genes not cleanly mapping to H37Rv, the protein-coding genes of 4,020 and 4,019 for RSA 114 and RSA 184, respectively, were predicted with Prodigal (9). Both strains had 45 tRNAs identified by tRNAscan-SE (11) and 3 rRNA genes predicted using RNAmmer (12). We also confirmed the experimental spoligotype predictions using a previously described computational spoligotyping approach (13).

Sequence reads were aligned to the *M. tuberculosis* H37Rv reference genome using BWA (14), and Pilon was used to identify variants. We detected a total of 797 and 734 nonsynonymous changes relative to H37Rv for RSA114 and RSA184, respectively. We also detected nonsynonymous changes in *rpoB* (S450L, I491F), *katG* (S315T), and *gyrA* (D94G), previously implicated in drug resistance. Interestingly, RSA114, which lacked known resistance-conferring *gyrA* mutations, had 14, 7, and 4 nonsynonymous changes in genes encoding efflux pumps (EPs), phthiocerol dimycocerosates (PDIMs) and type VII secretion systems (ESXs), respectively. Drug resistance in *M. tuberculosis* can be acquired through mutations in EPs that increase their activity to expel a broad spectrum of antibiotics (15), and ofloxacin-resistant strains, lacking DNA *gyrase* mutations, were found to overexpress EPs (16). In RSA114, we identified mutations within the EP-encoding genes Rv0987, Rv2039c, and Rv0402c that are predicted by PROVEAN (http://provean.jcvi.org/index.php), to impact efflux activity. ESX export enzymes are involved in the synthesis of PDIM proteins, which have a role in virulence (17), and are overexpressed in XDR TB strains, suggesting a contribution to this XDR-level drug resistance (18). Future functional studies are needed to determine the impact of these mutations on drug resistance.

### Nucleotide sequence accession numbers.

The whole-genome sequences for RSA114 and RSA184 have been deposited at NCBI GenBank under the accession numbers JKJF01000000 and JKQQ01000000, respectively.
